# Translation, cross-cultural adaptation and psychometric evaluation of the Portuguese version of the self-care in chronic obstructive pulmonary disease inventory

**DOI:** 10.1016/j.ijnsa.2025.100469

**Published:** 2025-12-09

**Authors:** Hélder Cunha, Patrício Costa, José Miguel Padilha

**Affiliations:** a Matosinhos Local Health Unit, Matosinhos, Porto, Portugal; b School of Medicine and Biomedical Sciences (ICBAS) – Porto University (UP), Porto, Portugal; c Center for Psychology at the University of Porto, Faculty of Psychology and Education Sciences, University of Porto, Porto, Portugal; d RISE-Health, Nursing School of Porto (ESEP), Porto, Portugal

**Keywords:** Chronic obstructive pulmonary disease, Middle-range theory, Nursing, Psychometrics, self-care, self-efficacy

## Abstract

**Background:**

Chronic obstructive pulmonary disease is a major global cause of death, marked by frequent exacerbations, high healthcare use, and reduced quality of life. Its progressive nature requires continuous self-care and symptom management. Understanding how individuals make self-care decisions is key to developing patient-centered nursing interventions. This study validates a theory-based instrument designed to assess core self-care domains in individuals living with chronic obstructive pulmonary disease, addressing gaps in existing tools that insufficiently capture behavioral aspects of disease management.

**Objective:**

To translate and adapt the Self-care in Chronic Obstructive Pulmonary Disease Inventory to the Portuguese context and to test its psychometric properties on a sample of patients with chronic obstructive pulmonary disease in Portugal.

**Design:**

A methodological study focused on the validation of an instrument.

**Setting(s):**

A Local Health Unit and its aggregated primary care centers from a municipality in the north region of Portugal.

**Participants:**

196 patients met the inclusion criteria and accepted to participate in the study.

**Methods:**

Construct validity was assessed using confirmatory factor analysis and hypothesis testing, while internal consistency reliability was evaluated using Cronbach’s alpha and McDonald’s omega.

**Results:**

A convenience sample of 196 chronic obstructive pulmonary disease patients was recruited from September 2023 to February 2024. The instrument consists of four scales: Self-Care Maintenance, Self-Care Monitoring, Self-Care Management and Self-Care Self-Efficacy. Confirmatory factor analysis was assessed on all four dimensions of the inventory producing acceptable model fit indices. The hypotheses presumed to evaluate the convergent and discriminant validity were confirmed. Internal consistency was adequate with Cronbach’s alpha values ranging from 0.673 to 0.893 across the four scales.

**Conclusions:**

The Portuguese version of the Self-Care in Chronic Obstructive Pulmonary Disease Inventory demonstrated acceptable psychometric properties. Some differences were observed compared to the original instrument. Further validation studies are needed to confirm its psychometric characteristics in the Portuguese population.

**What is already known**

• The Middle-Range Theory of Self-Care in Chronic Illness explains how individuals with chronic illnesses manage their self-care. It highlights the self-care maintenance, monitoring, and management processes, emphasizing the role of both patients and healthcare providers in improving health outcomes. It has proven to be a valuable framework for guiding nursing interventions to support self-care in individuals with chronic illnesses.
• In individuals with chronic obstructive pulmonary disease, effective self-care is crucial in maintaining disease stability and improving health-related quality of life.
• There is a lack of patient-reported outcome measures for chronic obstructive pulmonary disease that assess self-care behaviors. Based on a Middle-Range Theory, the Self-Care in Chronic Obstructive Pulmonary Disease Inventory has shown positive application outcomes in Italy, the United States, and China. However, its applicability in Portugal has yet to be tested.

Alt-text: Unlabelled box

**What this paper adds**

• The Portuguese version of the Self-Care in Chronic Obstructive Pulmonary Disease Inventory has shown good reliability and validity. It can be a fundamental tool to assess one’s capacity to perform chronic illness-related self-care behaviors.
• The Portuguese version of the instrument has revealed distinctive features compared to the original, suggesting that variations in healthcare contexts and cultural interpretations of self-care across countries may lead to differing self-care behaviors.
• To support wider use of the scale, it is essential to carry out cross-cultural adaptation across diverse populations in additional countries.

Alt-text: Unlabelled box

## 1 Background

Chronic Obstructive Pulmonary Disease (COPD) is a progressive and heterogeneous respiratory condition characterized by airflow limitation caused by structural changes and inflammation in the airways and/or alveoli (Global Initiative for Chronic Obstructive Lung Disease (GOLD), 2025). It is the fourth leading cause of death worldwide, accounting for 3.5 million deaths in 2021, primarily in low and middle-income countries (World Health Organization (WHO), 2024). Global prevalence was estimated at 10.3 %, with a projected increase due to population aging and rising smoking rates (Adeloye et al., 2022). Symptoms include chronic cough, sputum production, breathlessness, fatigue and psychological distress, significantly affecting quality of life and daily functioning (Holland, Dal Corso and Spruit, 2021). Pulmonary rehabilitation constitutes a cornerstone non-pharmacological intervention in the comprehensive management of COPD. While pharmacological treatments—such as bronchodilators and inhaled corticosteroids—primarily target symptom relief and reduction in the frequency of exacerbations, pulmonary rehabilitation is a core non-pharmacological strategy focused on improving functional capacity, reducing dyspnea, and enhancing quality of life through exercise, education, and promotion of self-care behaviors.

Self-care involves taking actions to maintain health, prevent illness, and manage chronic conditions. It encompasses monitoring one's symptoms, making informed treatment decisions, and engaging in behaviors that support overall health management (Riegel et al., 2019). Self-care in individuals with COPD has been shown to reduce perceived dyspnea, improve functional status, positively impact all-cause hospitalization, and enhance health-related quality of life (Schrijver et al., 2022). Effective prevention and management of acute exacerbations through adopting appropriate self-care behaviors are crucial for improving prognosis and quality of life. Recent literature emphasizes that healthcare professionals must move beyond traditional educational approaches to support individuals in developing sustainable self-care skills that adapt to the ongoing challenges of living with a chronic illness like COPD. Empowering patients to take an active and continuous role in their care is fundamental to building both knowledge and competence (Schrijver et al., 2022). Key areas to address include providing essential information about the disease, offering strategies to alleviate breathlessness, supporting smoking cessation, explaining the treatment regimen and inhaler use, and supplying an action plan for managing exacerbations (GOLD, 2025).

In order to prescribe tailored nursing interventions, considering each individual's unique characteristics, it is essential to understand the intentionality behind their self-care actions and to gather deeper insight into how they perceive and engage in self-care behaviors related to their chronic illness (Wang et al., 2020). Therefore, using robust instruments to assess self-care is crucial for accurately identifying individual needs, guiding clinical decision-making and developing effective, person-centered care plans.

Several validated tools are widely used in clinical practice to assess individuals with COPD, including the St. George’s Respiratory Questionnaire (Jones et al., 1992), COPD Assessment Test (CAT) (Jones et al., 2009), Clinical COPD Questionnaire (Van der Molen et al., 2003), and the London Chest Activity of Daily Living scale (Garrod, 2000). These instruments evaluate disease impact, symptom burden, health status, and activity limitations. However, until recently, no reliable, theory-driven tool existed to specifically evaluate self-care behaviors in this population.

The Self-Care in Chronic Obstructive Pulmonary Disease Inventory (SC—COPDI), developed by Matarese et al. (2020), is a Likert-type instrument that uses a scale ranging from one to five points, with responses gradually reflecting the item statements. It includes two components: the first part focuses on self-care behaviors, derived from the three categories of the Middle-Range Theory of Self-Care in Chronic Illness (Riegel, Jaarsma and Strömberg, 2012): self-care maintenance (behaviors aimed at improving well-being, preserving health, and maintaining physical and emotional stability), self-care monitoring (the process of routine, vigilant monitoring and surveillance of the body), and self-care management (the evaluation of changes in physical and emotional signs and symptoms to determine whether action is needed); the second part, the Self-Care Self-Efficacy Scale in COPD (SCES-COPD), which is not derived from the aforementioned Middle-Range Theory, assesses self-efficacy in self-care by quantifying the individual’s confidence in performing certain self-care behaviors, despite potential barriers (Matarese et al., 2020). The authors propose that, although not a component of self-care, self-efficacy is a key predictor of self-care behavior in previous studies and should, therefore, also be assessed (Riegel, Jaarsma and Strömberg, 2012; Vellone et al., 2016; Caruso et al., 2018).

The Self-Care in Chronic Obstructive Pulmonary Disease Inventory comprises 39 items across three components: self-care maintenance (13 items, 4 dimensions), self-care monitoring (8 items across 2 dimensions, plus 1 item on symptom recognition), self-care management (10 items, 3 dimensions) and the Self-Care Self-Efficacy Scale (7 items, 2 dimensions) (Matarese et al., 2020). SC—COPDI has demonstrated solid psychometric properties in Italy, the U.S., and China (Matarese et al., 2020; Bugajski et al., 2022; Wang et al., 2024).

Thus, our objective was to translate the SC—COPDI into Portuguese and evaluate its psychometric properties in a Portuguese sample of patients with COPD, specifically assessing its structural validity, construct validity through hypothesis testing, and internal consistency. This validation study will contribute to the cross-cultural applicability of the instrument and help improve the assessment of self-care behaviors in Portuguese-speaking populations with COPD.

## 2 Methods

### 2.1 Design

This methodological study was divided into two consecutive phases: (1) instrument translation and (2) evaluation of its psychometric properties through a cross-sectional study.

### 2.2 Translation and adaptation of the Self-care in Chronic Obstructive Pulmonary Disease inventory

The Self-Care in Chronic Obstructive Pulmonary Disease Inventory was translated according to the best practice guidelines for instrument translation and cultural adaptation outlined by Wild et al. (2005). These guidelines recommend the following ten steps: preparation, forward translation, reconciliation, backward translation, back-translation review, harmonization, cognitive debriefing, review of cognitive debriefing, finalization, and proofreading.

In the preparation phase a formal request for authorization to use and translate the instrument was made to the first author. The author was also invited to participate actively in the process. In the forward translation phase, the instrument was translated from English to Portuguese by two translators who worked independently: a Portuguese language professor with literary translation experience and no healthcare background and a healthcare professional from Portugal, living in an English-speaking country, fluent in English.

In the reconciliation phase, a consensus meeting was held by the researcher, the supervisor, and all translators from the previous phase to produce an alternative, reconciled version of the instrument. This version was then subjected to back-translation, the next step in the process. Two independent back-translations into the original language were carried out by bilingual healthcare professionals proficient in both English and Portuguese, with English as their native language. These translators had not participated in the earlier stages, were unaware of the study’s concepts and objectives, and had no access to the original instrument. The back-translations were then reviewed and compared by the researchers in order to address problematic items and refine the translation, as recommended in Step 5 (back-translation review). In the harmonization phase, a virtual meeting was convened by the investigators and both back-translators. During this session, a verbal back-translation of each item was requested to provide an additional layer of quality assurance. Careful attention was given to the semantic equivalence between the back-translated items and the original instrument, as well as to conceptual discrepancies previously identified. Potential solutions were discussed collaboratively, adhering to the methodological recommendations outlined by Wild et al. (2005). The outcome of this phase was a preliminary version of the instrument, reflecting the rigorous methodological trajectory undertaken throughout the translation and back-translation processes. During the cognitive debriefing phase, a pre-test was conducted with 20 native participants, aged between 55 and 86 years, representing a range of socioeconomic statuses and literacy levels, including individuals with and without chronic or respiratory conditions. A summary of the cognitive debriefing findings is presented in the Supplementary Material. Overall, the instrument was well comprehended; however, some suggestions for refinement emerged and were subsequently submitted to the original author for consideration in the following stage, review of cognitive debriefing and finalization.

The entire process was thoroughly reviewed in the last two stages, and the translation was carefully reread to identify any grammatical or typographical errors. At this stage, a final report detailing the translation methodology and key decisions was submitted to the original author. Following review and discussion, the final Portuguese version was approved (see Supplementary Material).

### 2.3 Psychometric evaluation

#### 2.3.1 Setting

In the psychometric evaluation study, patients diagnosed with COPD from a Local Health Unit and its aggregated primary care centers from a municipality in the north region of Portugal were recruited from September 2023 to February 2024.

#### 2.3.2 Participants

The required sample size was determined by the recommendation of Ribeiro (2010), which suggests including 5 to 10 individuals per variable under investigation. The sample size was adjusted based on the number of variables in the inventory to ensure adequate representation.

The inclusion criteria for the sample were as follows: individuals with a medically confirmed diagnosis of COPD; aged 18 years or older; receiving care for their chronic respiratory condition at the study setting; willing to participate in the study after reviewing the informational document and signing the informed consent form; and proficient in reading, writing, understanding, and speaking Portuguese. The exclusion criteria were: mental health conditions, intellectual disabilities, or significant cognitive and physical limitations. A non-probabilistic convenience sampling method was employed to recruit 196 patients who met the inclusion criteria and were accepted to participate in the study.

#### 2.3.3 Procedure

The study was introduced to the participants by the primary investigator and/or collaborators (registered nurses working with chronic respiratory patients), in a private setting (e.g., consultation room, home, office) to ensure participants' privacy. Individuals who agreed to participate completed questionnaires that covered sociodemographic information and the Self-Care in Chronic Obstructive Pulmonary Disease Inventory. It was estimated that each participant would take an average of 10 to 15 min to complete them. The investigator's presence during data collection was prioritized to address any questions or concerns that could arise. Collaborators were thoroughly trained on the instrument and its theoretical basis to support participants as needed.

#### 2.3.4 Variables and measures

The questionnaire contained two sections: (1) sociodemographic and clinical data, and (2) the instrument. Data were collected directly from participants and via the institution's health records. Sociodemographic variables included age, gender, marital status, education, employment, and co-habitation status. Clinical variables included years since diagnosis, airflow obstruction (GOLD stage), COPD group (A, B, or E), Modified Medical Research Council (mMRC) dyspnea scale, and COPD Assessment Test (CAT) score.

The Portuguese version of the Self-Care in Chronic Obstructive Pulmonary Disease Inventory comprises 39 items distributed across four distinct scales: Self-Care Maintenance (13 items), Self-Care Monitoring (8 items), Self-Care Management (10 items), and Self-Care Self-Efficacy (7 items). An additional item is included to differentiate between asymptomatic and symptomatic individuals by asking whether they have experienced symptoms in the past and how promptly they recognized them as indicative of a COPD exacerbation. Responses use a 5-point Likert scale, assessing either the frequency or likelihood of engaging in specific self-care behaviours (1 = never/not likely, 5 = always/very likely). Dimensional scores were computed using the formula: (mean – 1) × 25, yielding standardized scores ranging from 0 to 100. This transformation ensures score comparability across dimensions, despite the unequal number of items per scale. Item B9, designed to distinguish between asymptomatic and symptomatic patients, is excluded from the scoring algorithm, as it does not contribute to the quantitative assessment of self-care behaviors. Higher standardized scores reflect greater engagement in self-care behaviors.

The GOLD classification was used to assess the severity of COPD based on airflow limitation as measured by spirometry. It categorizes patients into four stages: Stage I (Mild) with Forced Expiratory Volume in one second (FEV₁) ≥ 80 % of the predicted value, Stage II (Moderate) with FEV₁ ≥ 50 % and < 80 %, Stage III (Severe) with FEV₁ ≥ 30 % and < 50 %, and Stage IV (Very Severe) with FEV₁ < 30 %. Regarding COPD groups, GOLD (2025) categorizes patients into three groups—A, B, and E—based on symptom severity (measured by mMRC and CAT) and exacerbation history per year (number of moderate exacerbations and/or hospitalization). Group A patients have low symptoms and exacerbation risk, Group B has high symptoms but low exacerbation risk, and Group E has both high symptoms and high exacerbation risk. The mMRC Dyspnea Scale (Mahler and Wells, 1988) was utilized to assess the severity of dyspnea and quantify the disability attributable to breathlessness. The scale ranges from 0 (indicating no limitation) to 4 (indicating very severe limitation). CAT score (Jones et al., 2009) was used to assess the impact of COPD symptoms on patients' overall health. It comprises eight items evaluating cough, phlegm, chest tightness, breathlessness, performance of housework activities, confidence, sleep, and energy levels. Each item is rated on a 6-point scale, ranging from 0 (no effect) to 5 (maximum effect). The total score ranges from 0 to 40, with scores of '0–10′ indicating slight impact, '11–20′ indicating moderate impact, '21–30′ indicating severe impact, and '31–40′ indicating very severe impact on health status.

### 2.4 Data analysis

Descriptive statistics were calculated for all variables, including frequencies, percentages, means, standard deviations (SD), and kurtosis and skewness coefficients where applicable. The following software were used to analyze data: JASP, IBM SPSS Amos v.26, and IBM SPSS Statistics v.26. The results are reported according to APA guidelines.

#### 2.4.1 Construct validity

Given the theoretical framework underlying the inventory, construct validity was assessed through confirmatory factor analysis and hypothesis testing, consistent with previous validation studies (Matarese et al., 2020; Wang et al., 2024). Each of the three dimensions of the SC—COPDI, as well as the SCES-COPD, were tested individually. Factor loadings greater than 0.30 were considered acceptable (Hair et al., 2010). Accounting for the non-normal distribution of the items, robust maximum likelihood estimation (MLR) was employed for parameter estimation to address the non-normality and potential heteroscedasticity of the data. Supplementary material includes confirmatory factor analysis model plots representing the various dimensions of the instrument. Model fit was assessed using the following indices: chi-squared (χ²) statistics, comparative fit index (CFI), goodness of fit index (GFI), and root mean square error of approximation (RMSEA). The following cut-off values were applied to evaluate model fit: CFI > 0.95, GFI > 0.90, and RMSEA values of < 0.05, indicating good fit, and between 0.05 and 0.08, indicating moderate fit (Hair et al., 2010). The χ^2^ statistics were computed and interpreted along with the mentioned indices. Given substantial correlations among the first-order factors, a second-order confirmatory factor model was evaluated to examine whether a higher-order latent construct could account for their shared variance (Hair et al., 2010).

Two primary hypotheses were formulated based on theoretical and empirical foundations to evaluate construct validity. First, it was hypothesized that the dimensions of self-care maintenance, self-care monitoring, and self-care management would exhibit moderate correlations, as they represent theoretically related but distinct components of the self-care process, as outlined in the Middle-Range Theory of Self-Care in Chronic Illness (Riegel, Jaarsma and Strömberg, 2012). This hypothesis supports the assessment of convergent validity. Second, it was posited that individuals with COPD presenting a higher symptom burden would report higher engagement in self-care behaviors, as symptom perception is a key driver of self-care activity aimed at mitigating symptom impact and enhancing health-related quality of life (Rivera et al., 2018). Specifically, participants with higher scores on the mMRC dyspnea scale, and COPD Assessment Test were expected to report significantly greater self-care across the respective dimensions. This latter hypothesis supports the assessment of discriminant validity.

To assess convergent validity, Pearson’s correlation coefficients were computed, with effect size interpretation based on Cohen’s (1988) thresholds: *r* < 0.30 (small), 0.30 ≤ *r* < 0.50 (moderate), and *r* ≥ 0.50 (strong). Group differences in self-care mean scores were analyzed using independent samples *t*-tests and one-way analysis of variance (ANOVA). Post hoc comparisons were conducted using Tukey’s Honestly Significant Difference (HSD) test. Effect sizes were calculated using Cohen’s d for *t*-tests, with thresholds of *d* < 0.20 (small), 0.20 ≤ *d* < 0.50 (medium), and *d* ≥ 0.80 (large), and eta squared (η²) for ANOVA, with values of η² < 0.01 (small), 0.01 ≤ η² < 0.06 (medium), and η² ≥ 0.14 (large) (Cohen, 1988).

#### 2.4.2 Internal consistency

Internal consistency was evaluated using Cronbach's alpha (α), a commonly used reliability coefficient that ranges from 0 to 1, with higher values indicating better consistency among scale items. Values above 0.80 are generally considered good, while values over 0.60 may be acceptable for exploratory studies or newly developed instruments (George and Mallery, 2010). To complement this, McDonald’s omega (ω) was also computed. Unlike alpha, which assumes equal item contributions (tau-equivalence), omega offers a more flexible and often more accurate estimate of reliability in the presence of unequal item loadings (Dunn, Baguley and Brunsden, 2014). Similar to α, ω values above 0.70 are typically interpreted as acceptable (Hayes and Coutts, 2020).

### 2.5 Ethical concerns

Data collection authorization was obtained from the Ethics and Health Committee of a Local Health Unit in northern Portugal. Participants were provided with an explanatory letter and informed consent form, ensuring voluntary participation and the right to withdraw without affecting their treatment. Anonymity was maintained by concealing identifiable information such as names and clinical records, with data used solely for statistical analysis.

## 3 Results

### 3.1 Participant characteristics

The sample comprised 196 participants with COPD with an average age of 71.4 years (*SD*=9.08). Of the participants, the majority were male, married, had only the first level of education, retired, and lived with a spouse/partner. Clinically, participants had an average of 9.23 years (*SD*=6.97) of COPD diagnosis. Obstruction levels varied, with 21.4 % having mild (GOLD 1), 46.9 % moderate (GOLD 2), and 5.6 % very severe (GOLD 4) obstruction. Regarding symptom severity and exacerbation history, most were in groups A and B, and 16.8 % were in Group E. Most participants had intermediate mMRC scores, with only 3.1 % reporting being too short of breath to leave the house. Additional information is provided in Table 1.

**Table 1:** Demographic and clinical profile of study participants (*n* = 196).

Items	N	( %)
**Age**, mean ± *SD* (range)	71,42 ± 9,08 (44–93)	
**Gender**		
Male	136	69.4 %
Female	69	30.6 %
**Marital status**		
Single	13	6.6 %
Married	133	67.9 %
Divorced	16	8.2 %
Widowed	34	17.3 %
**Education**		
Illiteracy	6	3.1 %
Elementary	117	59.7 %
Second cycle	18	9.2 %
Third cycle	16	8.2 %
High School	24	12.2 %
University	15	7.7 %
**Employment Status**		
Employed	24	12.2 %
Unemployed	12	6.1 %
Retired	159	81.1 %
Other	1	0.5 %
**Living arrangements**		
Alone	35	17.9 %
With Spouse/Partner	99	50.5 %
With Spouse/Partner and children	37	18.9 %
With children	15	7.7 %
With friends	2	1 %
With other relatives	8	4.1 %
**COPD years**, mean ± *SD* (range)	9,23 ± 6,97 (1–40)	
**Degree of obstruction**		
GOLD 1	42	21.4 %
GOLD 2	92	46.9 %
GOLD 3	51	26 %
GOLD 4	11	5.6 %
**COPD Groups**		
A	84	42.9 %
B	79	40.3 %
E	33	16.8 %
**mMRC**		
0	21	10.7 %
1	81	41.3 %
2	57	29.1 %
3	31	15.8 %
4	6	3.1 %
**CAT**		
≤ 10	67	34.2 %
11–20	47	24 %
21–30	37	18.9 %
31–40	6	3.1 %
Missing data	39	19.9 %

Abbreviations: SD: standard deviation; COPD: Chronic Obstructive Pulmonary Disease; GOLD: Global Initiative for Chronic Obstructive Lung disease – degree of obstruction; mMRC: modified Medical Research Council dyspnoea scale; CAT: COPD Assessment Test;.

### 3.2 Construct validity

#### 3.2.1 Self-care maintenance dimension

Confirmatory factor analysis of the original four-factor model for self-care maintenance (13 items) showed acceptable fit: χ²(59) = 89.28, *p* = .007; CFI = 0.938; GFI = 0.981; RMSEA = 0.051 (90 % CI [.03, 0.07], *p* = .439). Additional evaluated fit indices for all model dimensions are provided in Table 2. Most items demonstrated adequate loadings (> 0.30), except item A9 (“Participation in social activities”) and item A10 (“Annual influenza vaccination”), which fell below threshold.These items also displayed skewed distributions and ceiling effects, particularly A10 and A11 (“Medication adherence”), suggesting reduced discriminatory capacity. A significant residual covariance between items A7 and A8 (both related to exercise) was theoretically justified and retained. The strongest factor correlation was observed between disease prevention and treatment adherence behaviors (*r* = 0.736, *p* < .001), aligning with theoretical expectations.

**Table 2:** Evaluated Fit Indices for All Model Dimensions.

Scale	*χ2*	*df*	P(*χ2)*	CFI	GFI	RMSEA [90 % CI] (p)
Self-care maintenance	89.28	59	0.007	0.938	0.981	0.051 [0.03, 0.07] (0.439)
Self-care monitoring	42.51	18	< 0.001	0.998	0.997	0.084 [0.05, 0.12] (0.045)
Self-care management	45.18	30	0.037	0.999	0.999	0.057 [0.01, 0.09] (0.337)
Self-care self-efficacy	26.96	12	0.008	0.996	0.999	0.080 [0.04, 0.12] (0.102)

Abbreviations: CFI: comparative fit index; CI: confidence interval; df: degree of freedom; RMSEA: root mean square error of approximation; GFI: Goodness of fit index; χ2: chi-square.

#### 3.2.2 Self-care monitoring dimension

The two-factor model for monitoring activities (8 items) demonstrated good fit: χ²(18) = 42.51, *p* < .001; CFI = 0.998; GFI = 0.997; RMSEA = 0.084 (90 % CI [.05, 0.12], *p* = .045). Factor loadings ranged from 0.39 to 0.97 and were all statistically significant. A residual covariance between items B5 and B6 (both related to nocturnal symptoms) was allowed, improving model fit. The two self-care monitoring factors were strongly correlated (*r* = 0.736, *p* < .001).

#### 3.2.3 Self-care management dimension

Initial model testing for the three-factor structure (10 items) showed a poor model fit: [χ²(30) = 205.49, *p* < .001; CFI = 0.999; GFI = 0.999; RMSEA = 0.173 (90 % CI [0.15, 0.20], *p* < .001)]. We explored the cause of the initial model misfit and found that although modifications such as reassigning items or adjusting residual covariances improved fit statistically, they lacked theoretical justification.

Upon further reflection, we noted that during translation procedures, the original author instructed us to remove the option to skip Section C, requiring all participants to complete the entire inventory regardless of whether they had symptoms. We then tested the model with only symptomatic participants (*n* = 155), as in the Italian validation, yielding an acceptable fit: [χ²(30) = 45.18, *p* = .037; CFI = 0.999; GFI = 0.999; RMSEA = 0.057 (90 % CI [0.015, 0.090], *p* = .337)]. We hypothesize that this change in procedure contributed to the initial misfit. For example, in items C9 or C10, where there is no option to respond 'not applicable', asymptomatic individuals may naturally indicate that they do not sit to perform household tasks or to take a shower. Consequently, they might choose a 1 (unlikely) on the Likert scale, which does not imply poor problem-solving skills.

#### 3.2.4 Self-care self-efficacy

The two-factor model for self-efficacy (7 items) showed satisfactory fit: χ²(12) = 26.96, *p* = .008; CFI = 0.996; GFI = 0.999; RMSEA = 0.080 (90 % CI [.04, 0.12], *p* = .102). Factor loadings were strong and significant (0.62 to 0.94). A residual covariance between items D2 and D4, both related to adherence behaviors was allowed, resulting in improved model fit. This pattern mirrors findings from the original validation study (Matarese et al., 2020). All descriptive statistics and factor loadings for the Self-care scales are presented in Table 3.

**Table 3:** Descriptive statistics and factor loadings for the Self-Care Scales.

Items	Mean (*SD*)	Skewness	Kurtosis	Loading
**Self-care maintenance (A)**	**64.25 (14.75)**			
Factor 1: Disease prevention behaviors				
1) Avoid people with cold or flu	3.66 (1.42)	−0.64	−0.96	.56
2) Move away from places where someone is smoking	3.60 (1.57)	−0.56	−0.28	.73
3) Avoid contact with sprays, paints, solvents, and dust	3.74 (1.43)	−0.76	−0.78	.57
12) Protect mouth/nose in cold air	2.20 (1.37)	1.37	−0.29	.57
Factor 2: Improving breathing behaviors				
(4) Keep lungs free by coughing or with deep breathing	3.60 (1.05)	−0.15	−0.73	.57
(5) Pause during daily activities	3.43 (1.34)	−0.32	−1.05	.56
(6) Use abdominal or blurred lips breathing	2.91 (1.29)	.07	−0.94	.71
Factor 3: Physical activities promotion behaviors				
(7) Exercise regularly (walking, cycling, swimming, etc.)	3.36 (1.39)	−0.32	−1.10	.71
(8) Do arm exercises	2.87 (1.43)	.17	−1.25	.85
(9) Engage in social activities	3.20 (1.62)	−0.17	−1.58	.21
Factor 4: Treatment adherence behaviors				
(10) Get a flu vaccine every year	4.72 (0.90)	−3.35	10.38	.30
(11) Take the medicines as prescribed	4.65 (1.10)	−3.50	11.59	.61
(13) Make regular visits to health-care provider for checkups	4.41 (1.04)	−1.69	1.90	.62
**Self-care monitoring (B)**	**41.5 (28)**			
Factor 1: Respiratory symptom monitoring behaviors				
1) Monitor the increase of phlegm	2.95 (1.81)	−0.50	−1.07	.97
2) Monitor the color change of phlegm	3.12 (1.83)	−0.61	−0.99	.96
3) Monitor the increase of coughing	3.15 (1.73)	−0.64	−0.75	.88
4) Monitor for an increase in breathlessness or whistles	3.78 (1.51)	−1.35	1.01	.59
Factor 2: Extra-respiratory symptom monitoring behaviors				
(5) Monitor waking up during the night because of trouble breathing	1.41 (1.81)	.94	−0.60	.66
(6) Check struggle to fall asleep due to trouble breathing	1.22 (1.71)	1.14	−0.11	.60
(7) Monitor getting tired when doing something	3.46 (1.52)	−0.86	−0.04	.66
(8) Check for side effects of inhaled medications	2.16 (1.49)	.66	−0.69	.39
**Self-care management (C)**	**53 (25.75)**			
Factor 1: Autonomous behaviors				
(1) Talk to health-care provider for problems with prescriptions	3.48 (1.49)	−0.77	−0.11	.77
(2) Go to health-care provider for health problems	3.93 (1.19)	−0.63	−0.80	.80
(8) Modify therapy as recommended when symptoms worsen	2.73 (1.78)	−0.01	−1.43	.42
Factor 2: Consulting behaviors				
(3) Speak to health-care provider if breathlessness increases	3.85 (1.27)	−0.67	−0.68	.87
(4) Speak to health-care provider if coughing increases	3.14 (1.63)	−0.51	−0.77	.96
(5) Speak to health-care provider if sputum increases	3.16 (1.70)	−0.55	−0.87	.88
(6) Speak to health-care provider if sputum changes color	3.13 (1.70)	−0.52	−0.90	.89
(7) Speak to health-care provider for side effects of inhaled medications	2.71 (1.63)	.07	−1.20	.65
Factor 3: Problem-solving behaviors				
(9) Sit doing housework when breathlessness occurs	2.82 (1.56)	.22	−1.41	.87
(10) Sit when showering in case of breathlessness	2.27 (1.57)	.80	−0.94	.98
**Self-care self-efficacy (D)**	**64.25 (21.75)**			
Factor 1: Symptom management				
(1) Prevent the onset of symptoms	3.14 (1.17)	−0.06	−0.48	.75
(5) Recognize the symptoms of an exacerbation	4.23 (0.92)	−0.63	−1.14	.75
(6) Do something to relieve symptoms	3.53 (1.11)	−0.27	−0.49	.90
(7) Assess the effectiveness of behaviors performed to relieve symptoms	3.33 (1.16)	−0.26	−0.45	.94
Factor 2: Treatment adherence				
(2) Follow the therapeutic advice	3.89 (1.03)	−0.34	−0.95	.73
(3) Continue to check symptoms	3.53 (1.12)	−0.21	−0.62	.97
(4) Take medicines following the instructions given	4.23 (0.92)	−0.63	−1.14	.63

Abbreviations: SD: standard deviation;.

### 3.3 Hypothesis testing (convergent and discriminant validity)

Bivariate correlation analyses examined the relationships among the instrument’s dimensions and clinical variables (Table 4). Statistically significant positive correlations were found between Maintenance and Monitoring, r(196) = 0.33, *p* < 0.01; Maintenance and Management, r(196) = 0.48, *p* < 0.01; and Monitoring and Management, r(196) = 0.58, *p* < 0.01, indicating moderate to strong interrelations among self-care domains. Associations with age and time since diagnosis were weak (*r* < 0.30), suggesting minimal influence of these variables.

**Table 4:** Correlations among self-care scores and clinical disease severity variables assessed via variance analysis.

	Self-care Maintenance mean (*SD*)	Self-care Monitoring mean (*SD*)	Self-care Management mean (*SD*) (*n* = 155)
**GOLD**			
1	64.99 (15.80)	31.62 (30.74)a	42.16 (28.03)a
2	62.44 (14.68)	39.71 (25.60)a	49.50 (22.20)
3	68.91 (12.06)	48.96 (28.58)	58.10 (25.20)
4	62.97 (17.01)	58.81 (18.43)b	65 (16.07)b
**mmRC**			
0	65.33 (17.38)	25 (38.31)a	45.57 (30.63)
1	62.39 (13.82)	33.60 (23.27)a	44.53 (21.52)a
2	64.88 (15.67)	45.45 (26.07)	52.41 (23.68)
3	69.66 (9.90)	62.5 (20.48)b	66.76 (24.21)b
4	66.49 (21.04)	58.33 (23.52)b	63.80 (20.12)
**CAT**			
0–10	62.87 (12.77)	25.19 (25.86)	40.32 (21.10)
>11	67.09 (12.89)⁎⁎	47.85 (21.55)⁎⁎	55.18 (21.96)⁎⁎
**Self-care Maintenance**	-	.33*	.48*
**Self-care Monitoring**	-	-	.58*

Note: Means in columns identified with superscript letters a and b significantly differ for *p*<.05.

Abbreviations: GOLD: Global Initiative for Chronic Obstructive Lung disease – degree of obstruction; mMRC: modified Medical Research Council dyspnoea scale; CAT: COPD Assessment Test; SD: standard deviation;.

⁎ *p* < .01

⁎⁎ *p* < .05

Discriminant validity was assessed by comparing mean self-care scores across the subgroups stratified by CAT and mMRC levels. It was hypothesized that individuals reporting higher symptom burden and dyspnea would exhibit significantly elevated scores in all self-care domains, reflecting greater engagement in self-care behaviors.

Individuals with greater symptom burden demonstrated higher self-care behaviors, specifically in maintenance, management, and monitoring, ordered by effect size. Moreover, those with a higher mMRC score exhibited significantly greater self-care management and monitoring, although no statistically significant differences were observed in self-care maintenance.

### 3.4 Internal consistency

Internal consistency for the self-care maintenance domain was modest (α = 0.673; ω = 0.680), mainly due to the suboptimal performance of items A10 and A11 in the treatment adherence factor. In the self-care monitoring domain, internal consistency was satisfactory (α = 0.821; ω = 0.812). For the self-care management dimension, internal consistency was good (α = 0.854; ω = 0.837). Finally, the self-care self-efficacy dimension demonstrated excellent internal consistency (α = 0.893; ω = 0.900).

## 4 Discussion

The reporting of this study was guided by the Strengthening the Reporting of Observational studies in Epidemiology (STROBE) checklist (von Elm et al., 2007). The psychometric evaluation of the Self-Care in Chronic Obstructive Pulmonary Disease Inventory supports its validity and reliability in the Portuguese context. We employed a confirmatory factor analytic approach to examine the dimensional structure to assess construct validity. The original four-factor model of the self-care maintenance domain showed good fit. Most items had acceptable loadings (>0.30) though items A9 (“Participation in social activities”) and A10 (“Annual influenza vaccination”) fell below this threshold, suggesting limited relevance. Item A9 had similarly low loadings in previous studies (Matarese et al., 2020), highlighting a potential need for revision to improve conceptual alignment.

Item A10, concerning influenza vaccination, reflects a critical health behavior; however, its low factor loading may stem from its differential relevance across respondents. This variability likely resulted in restricted response variance, thereby attenuating its explanatory power within the model (Kline, 2016). Furthermore, both A10 and A11 showed evidence of a ceiling effect, a common limitation in self-reported behavioral assessments (Streiner, Norman and Cairney, 2015). In such cases, individuals who consistently perform a behavior may report little to no variation, constraining the item’s discriminatory capacity. To address this, future iterations of the instrument might consider incorporating items that explore barriers or situational factors influencing adherence, rather than relying solely on behavioral frequency. This approach would enable a more nuanced assessment and could enhance the instrument’s overall psychometric robustness. In Portugal, annual influenza vaccination is strongly recommended for individuals with chronic respiratory conditions, including COPD, and is provided free of charge through the National Health Service. Consequently, vaccine uptake in this population is typically high due to both accessibility and consistent reinforcement by healthcare providers. This widespread adherence likely accounts for the elevated mean scores and limited response variability observed for item A10, contributing to the ceiling effect. These findings align with national public health strategies to reduce the risk of exacerbations and complications from respiratory infections in high-risk groups (Direção-Geral da Saúde, 2023).

The factor with the lowest mean scores was 'physical activity promotion behaviors,' with a mean of 3.14 (*SD* ± 1.08). Among the items within this factor, item A8 – 'I exercise my arms at least three times a week' – reported the lowest frequency, with a mean score of 2.87 (*SD* ± 1.43). This could be attributed to participants not perceiving the need to engage in this activity intentionally, as they may already incorporate upper limb movements into their daily tasks, such as house cleaning, cooking, or other routine activities, and consider these actions sufficient. Supporting this observation, McKeough et al. (2016), in their systematic review, highlight that individuals with COPD often experience dyspnea and arm fatigue during upper limb activities, which may reduce participation in exercises targeting the upper limbs. Another item within the same factor that warrants further interpretation is Item A9 – *“Participation in social activities.”* Although distinct from the physical behaviors captured by other items in the factor, its results may also reflect how cultural norms shape the perception and reporting of self-care behaviors. Among older Portuguese adults, informal or routine interactions may not be perceived as “social activities” in the sense implied by the item. For example, going to the local coffee shop (*café*) is a common and culturally embedded habit, particularly among older individuals, yet many may not consider this a form of social participation—despite its important role in maintaining regular social contact. In traditional Portuguese communities, social engagement often occurs in informal, intergenerational contexts, such as family gatherings, religious events, or casual neighborhood conversations, which may not be consciously recognized or reported as structured social involvement. Additionally, cultural values emphasizing modesty and privacy may contribute to underreporting of such activities. These contextual factors highlight the importance of interpreting item-level results within the sociocultural environment in which self-care behaviors take place. Regarding the covariance between factors, the highest was found between Factor 1 and Factor 4, r(196) = 0.736, *p* < .001), representing disease prevention behaviors and treatment adherence behaviors, respectively, which aligns with the theoretical framework. In the context of chronic diseases like COPD, behaviors aimed at preventing disease exacerbations (e.g., avoiding triggers, regular exercise) often overlap with those that are related to adherence to medical treatments (e.g., taking prescribed medications and following healthcare provider recommendations). These two factors are both integral to managing the disease and improving the patient's health outcomes (GOLD, 2025).

The original two-factor structure was confirmed to have an adequate model fit in the self-care monitoring dimension. Notably, this dimension exhibited the lowest mean score among all dimensions in the instrument (*M* = 2.66, *SD* ± 1.12), indicating that participants engaged less consistently in symptom monitoring and recognition behaviors. As suggested by the theoretical framework underpinning the instrument, self-care is influenced by multiple interrelated factors (Riegel et al., 2012, 2019). In the Portuguese context, particularly among older adults, there is often a strong reliance on healthcare professionals for disease management, which may reduce the perceived need for individual monitoring or proactive self-care behaviors. Cultural norms may also play a role, as some individuals may not perceive symptom tracking or self-assessment as a personal responsibility in managing chronic conditions like COPD. This perspective may influence how patients respond to symptoms when they occur. As highlighted by Riegel et al. (2022), patients who are able to recognize their symptoms are more likely to engage in autonomous self-care—behaviors initiated independently in response to symptoms, such as sitting down to rest. In contrast, those who struggle with symptom recognition may bypass autonomous actions and move directly from monitoring to consulting a healthcare provider. Furthermore, symptom perception—shaped by psychological, cognitive, and sociocultural factors— often plays a dual role in self-care: it can both prompt and result from self-care behaviors. For instance, individuals who frequently experience symptoms may become more vigilant in perceiving changes in their condition, whereas those experiencing depression, cognitive impairment, or social isolation may have reduced motivation to engage in such monitoring behaviors (Auld et al., 2018; Riegel et al., 2019, 2022).

Additionally, varying levels of health literacy—particularly among participants with lower educational attainment—could impact their understanding of the importance of regular monitoring or their confidence in performing such tasks. On a systemic level, limited emphasis on patient education and empowerment in routine primary care settings may also contribute to reduced engagement with self-monitoring practices. Nevertheless, within Factor 1 (monitoring respiratory symptoms), the mean score was 3.25 (*SD*±1.45), suggesting more frequent monitoring of symptoms like sputum and cough, which are common during exacerbations (GOLD, 2025). Despite this, higher self-care scores would be desirable, as these symptoms are often part of daily life for many with COPD (GOLD, 2025). Clinical factors, such as the severity and frequency of prior exacerbations, may influence how closely individuals monitor these symptoms (Riegel et al., 2019). Item B4 (“Check for an increase in shortness of breath or wheezing”) showed a higher mean score (*M* = 3.78, *SD*±1.51), indicating that participants placed greater importance on dyspnea and wheezing as key signs of worsening respiratory health, aligning with findings from different studies (Miravitlles and Ribera, 2017; Vogelmeier et al., 2020; Melhem et al., 2021). Factor 2, which focuses on monitoring extra-respiratory symptoms, showed very low mean scores (*M* = 2.07, *SD*±1.16). Only fatigue during activity (item B7) was monitored consistently (*M* = 3.46, *SD*±1.52). Other items, such as night-time breathing difficulties and sleep disturbances, scored notably lower on our sample, suggesting a lack of awareness or perceived relevance of these symptoms. Despite this variability between factors, there was still a significant covariance between them (r(196) = 0.69, *p* < .001), supporting the theoretical premise that symptom clusters—especially in chronic diseases like COPD—frequently co-occur and influence each other (Houben-Wilke et al., 2024).

Within the self-care management dimension, the original model structure was tested exclusively among symptomatic participants (*n* = 155), defined as those who did not select ‘not applicable´ for item B9, which assesses both the presence of symptoms and the promptness of their recognition. The reason for it was already explained above. Nonetheless, significant residual covariances were identified between certain items, prompting adjustments that improved the model fit. For example, items C5 and C6, which assess changes in sputum quantity and color, respectively, demonstrated strong covariance, an association that is theoretically sound given the clinical interrelation of these symptoms. A similar pattern was reported in the Italian validation study (Matarese et al., 2020). Additionally, items C9 (“I sit down while doing housework when I feel short of breath”) and C10 (“When I feel short of breath while showering, I sit on a chair or another support”) also presented significant residual covariance. As both relate to the compensatory behavior of sitting in response to dyspnea during daily activities, their statistical relationship is theoretically congruent. Item C8, which refers to the autonomous decision to adjust medication in response to exacerbations, exhibited the lowest factor loading. This likely reflects the clinical complexity and perceived risk of such behavior. Some individuals with COPD—particularly those recently diagnosed or with milder disease—may not yet have developed the self-management competencies or confidence to make treatment adjustments independently. Additionally, many patients may not have been explicitly advised or empowered to make such decisions by their healthcare providers. This issue may be especially relevant in the Portuguese healthcare context, where medical authority tends to remain clinician-centered, and patient education is often limited—particularly among older adults and those with lower levels of formal education. This finding aligns with Riegel’s Middle-Range Theory of Self-Care of Chronic Illness, which highlights age, education, cognitive function and access to care as critical contextual factors influencing self-care behaviors (Riegel et al., 2012). In our sample, the predominance of older adults with limited formal education may have contributed to lower engagement in autonomous management, particularly in complex behaviors such as medication adjustment during exacerbations. While our study did not assess cognitive status, we acknowledge that cognitive impairment may partly explain lower scores on items requiring independent decision-making, such as medication adjustments. Future validation studies should consider incorporating brief cognitive screening tools to explore this relationship more directly. From a clinical perspective, the presence of cognitive vulnerability may necessitate greater involvement of caregivers or family members in supporting symptom response and self-care management. Integrating caregivers into care planning may be especially important in promoting safe and effective disease management for cognitively at-risk individuals.

Moreover, the use of rescue medication packs for COPD management is not universally adopted. While it is a guideline-recommended practice in the United Kingdom’s National Institute for Health and Care Excellence (NICE, 2019) it remains less common in other healthcare systems.

Key concerns include the risk of incorrect self-diagnosis, inappropriate or premature use, delayed medical consultation, and adverse outcomes such as antimicrobial resistance and steroid-related complications. The effectiveness of rescue packs also relies heavily on adequate patient education, which is often insufficient. (Lenferink et al., 2017; NICE, 2019; GOLD, 2025).

Finally, with respect to self-care self-efficacy, the original two-factor model was tested and demonstrated satisfactory model fit indices. The analysis revealed strong and statistically significant item-to-factor loadings, ranging from 0.62 to 0.94, indicating a robust association between individual items and their respective latent constructs.

During the hypothesis testing phase, the intercorrelations among the self-care dimensions were examined, providing empirical support for the theoretical framework underlying the instrument. The most robust association was observed between the monitoring and management dimensions, r(196) = 0.579, *p* < .01), corroborating the conceptual premise that symptom surveillance and recognition are antecedents to the decision-making processes involved in self-care management. These cognitive processes, although modulated by individual-level factors (e.g., experience, cognitive function, motivation), typically guide adaptive behavioral responses aimed at re-establishing physiological homeostasis (Riegel, Jaarsma and Strömberg, 2012). The magnitude of the correlations identified in this sample was comparable to or marginally higher than those reported in the Italian validation study, and substantially greater than those found in the Chinese cohort, where a negative correlation between maintenance and management behaviors was documented (Matarese et al., 2020; Wang et al., 2024). These findings highlight potential cultural or contextual influences on the structure and expression of self-care behaviors across populations.

Self-efficacy demonstrated positive correlations with all dimensions of the inventory; however, these correlations were notably weaker for the monitoring dimension. This finding indicates limited confidence among participants in their ability to engage in surveillance-related self-care behaviors. Such behaviors often require nuanced symptom interpretation and are heavily influenced by individuals’ experiential knowledge, illness representation, and familiarity with disease-specific manifestations—factors that may not be well-developed in all patients (Riegel et al., 2019).

To evaluate discriminant validity—specifically, whether a higher symptom burden correlates with increased self-care engagement—our analysis revealed that individuals with COPD Assessment Test (CAT) scores above 10 exhibited significantly higher mean scores across all self-care dimensions. Similarly, participants classified as Global Initiative for Chronic Obstructive Lung Disease (GOLD) stage 4 and those with elevated Modified Medical Research Council Dyspnea Scale (mMRC) scores (grades 3 and 4) demonstrated greater engagement in the monitoring and management dimensions, with statistically significant correlations, thereby supporting our hypothesis, and aligning with prior validation studies (Matarese et al., 2020; Wang et al., 2024). Notably, no significant associations were found between the degree of airway obstruction or perceived dyspnea and engagement in maintenance behaviors. This may reflect the fact that such behaviors—particularly those related to physical activity and social participation—require functional capacity and quality of life that may be compromised in more symptomatic individuals, thus limiting their ability to maintain consistent health-promoting routines. Factors such as physical limitations, psychological distress, and lack of motivation have been identified as barriers to physical activity in COPD patients (Cox et al., 2017; Rochester et al., 2018).

The Self-Care in Chronic Obstructive Pulmonary Disease Inventory is based on a well-established theoretical construct and a widely recognized conceptual model, demonstrating acceptable psychometric properties in a Portuguese population. Accordingly, although the instrument generated acceptable measurement outcomes, certain structural discrepancies emerged when compared with previously validated models. This phenomenon is consistent with findings reported in the literature, where variations in factor structure and item performance have been observed during the cross-cultural adaptation and validation of self-care instruments in chronic disease populations (Jaarsma et al., 2009; Vellone et al., 2014, 2015).

### 4.1 Limitations

Although this study provides important contributions to the understanding of self-care competencies in individuals with COPD, several limitations should be acknowledged.

This study employed a convenience sampling strategy, with participants recruited from a local health unit and affiliated primary care centres in the northern region of Portugal. While this facilitated recruitment and allowed for initial testing of the instrument in a real-world clinical setting, it introduces limitations regarding the representativeness and generalizability of the findings. The sample demonstrated a gender imbalance (69.4 % male) and a predominance of retired individuals (81.1 %), which may not reflect the broader COPD patient population in Portugal or other Portuguese-speaking countries. Additionally, the limited geographical coverage may restrict the applicability of the results to populations with different regional, cultural, or healthcare system characteristics. These limitations should be considered when interpreting the findings. Future research should consider employing multi-centre, stratified sampling strategies to ensure broader regional representation and greater diversity in terms of gender, age, socioeconomic background, and disease severity. Such approaches would enhance sample diversity and improve the external validity of the instrument. Additionally, although the targeted sample size was achieved, a larger and more diverse sample would have been preferable to enhance statistical power and improve the robustness of the model fit indices. Finally, predictive validity and test–retest reliability were not assessed in the present study. We recommend that future research address both in order to establish the instrument’s temporal stability and its capacity to predict meaningful clinical outcomes (Hair et al., 2010). Given the cross-sectional nature of the data, these properties could not be evaluated. Future research should address this gap by employing longitudinal designs to assess the instrument’s ability to detect changes over time and ensure score reproducibility under stable clinical conditions. We also recommend that future studies assess measurement invariance using multi-group confirmatory factor analysis to determine whether the scale functions equivalently across subgroups such as GOLD stages or healthcare-use profiles (Hair et al., 2010). This would help confirm the instrument’s fairness and applicability across diverse clinical populations.

### 4.2 Implications for practice

The Self-Care in Chronic Obstructive Pulmonary Disease Inventory provides clinically meaningful insight into the self-care behaviors of individuals with COPD and holds significant potential to inform nursing and clinical practice across care settings. Its three sub-scales—Self-Care Maintenance, Monitoring, and Management—along with their respective sub-factors, enable the identification of specific areas where patients may benefit from targeted, personalized interventions.

For instance, within the Self-Care Maintenance domain, low scores in “disease prevention behaviors”(e.g., avoiding triggers) may reflect gaps in patient knowledge or risk perception, indicating the need for focused education on modifiable risk factors, while deficits in “physical activity promotion behaviors” may indicate the value of tailored interventions such as pulmonary rehabilitation, motivational support strategies, or structured activity programs designed to accommodate functional limitations commonly experienced by this population. In the Self-Care Monitoring domain, low engagement in “respiratory symptom monitoring*”* may indicate difficulties in recognizing or interpreting early signs of exacerbation. This highlights the potential utility of structured symptom-tracking tools, nurse-led monitoring protocols, or digital health interventions aimed at enhancing symptom awareness.

Within the Self-Care Management domain, low engagement in “autonomous behaviors”—such as adjusting medications in response to symptom changes—may reflect the need for individualized action plans, use of teach-back methods to reinforce understanding, or scenario-based coaching to build decision-making confidence. When gaps are identified in “consulting behaviors” (e.g., seeking professional help during symptom worsening), interventions might focus on strengthening communication skills, providing clear symptom-action thresholds, and improving care access through telehealth or scheduled follow-ups.

The instrument’s design supports practical applications throughout the COPD care continuum. In primary care, it can help identify individuals with low self-care engagement who may benefit from targeted support or education. In pulmonary rehabilitation settings, it may be used to evaluate behavioral change following intervention. In hospital discharge planning, the Self-Care in Chronic Obstructive Pulmonary Disease Inventory can inform post-discharge follow-up by identifying individual self-care needs.

These domain-specific insights allow care teams to tailor self-management support to each patient’s profile, contributing to more proactive, efficient, and patient-centered care. Although formal cut-off scores are not yet established, preliminary use of sub-scale quartiles may help flag individuals with limited self-care engagement, guiding early intervention strategies and enhancing the delivery of personalized care.

## 5 Conclusions

The development of self-care competencies in individuals with chronic diseases, such as COPD, can be significantly influenced by nursing interventions. Providing tools that streamline the decision-making process is essential for designing targeted interventions that improve patients' quality of life and overall well-being.

Effective COPD management requires a comprehensive approach that integrates both pharmacological and non-pharmacological strategies, such as respiratory rehabilitation, aimed at promoting assertive self-care behaviors. In this context, Riegel and colleagues’ (2012) self-care theory offers a robust conceptual framework, identifying the core competencies individuals need to maintain, monitor, and manage chronic illness. Additionally, self-efficacy—the belief in one’s ability to perform specific actions—is a key factor in adherence to essential self-care behaviors, making it a vital concept to explore. Despite the growing body of research on chronic respiratory diseases, there has been a lack of systematic tools to assess self-care competencies in this population.

This instrument offers clinically relevant insight into self-care behaviors among individuals with COPD and can be effectively used to support assessment and care planning across diverse healthcare settings. For example, it may assist primary care providers in identifying patients with low self-care engagement who could benefit from targeted education or support programs. In respiratory rehabilitation settings, the instrument may help evaluate changes in behavior following intervention. It could also inform discharge planning by identifying specific self-care needs and guiding follow-up strategies for hospitalized patients. These applications contribute to more individualized and proactive care throughout the COPD care continuum.

Building on this clinical relevance, the Portuguese version of the Self-Care in Chronic Obstructive Pulmonary Disease Inventory demonstrated satisfactory psychometric properties in a sample of COPD patients in Portugal. While these findings support its use in both clinical and research contexts, further studies are needed to address the limitations identified and confirm its applicability in broader and more diverse populations. Continued validation work will help refine the instrument’s use across different healthcare settings and strengthen its cross-cultural relevance.

## Funding

This research did not receive any specific grant from funding agencies in the public, commercial, or not-for-profit sectors.

## CRediT authorship contribution statement

**Hélder Cunha:** Writing – original draft, Software, Resources, Project administration, Methodology, Investigation, Formal analysis, Data curation, Conceptualization. **Patrício Costa:** Writing – review & editing, Supervision, Software, Methodology, Data curation. **José Miguel Padilha:** Writing – review & editing, Supervision, Software, Methodology, Investigation, Formal analysis, Data curation, Conceptualization.

## Declaration of competing interest

The authors declare that they have no known competing financial interests or personal relationships that could have appeared to influence the work reported in this paper.

## Data Availability

The data that support the findings of this study are not publicly available due to privacy and ethical restrictions involving sensitive participant information. However, the data may be made available from the corresponding author upon reasonable request and with appropriate institutional approval.

## Appendix Supplementary materials

Image, application 1
