# Supplementary material for: Translation, cross-cultural adaptation and psychometric evaluation of the Portuguese version of the self-care in chronic obstructive pulmonary disease inventory
Source: Int J Nurs Stud Adv. 2025 Dec 9;10:100469. doi: 10.1016/j.ijnsa.2025.100469 (PMC12771086; doi:10.1016/j.ijnsa.2025.100469)
Supplement: Supplementary file 1 [file mmc1.docx]

**SUPPLEMENTARY MATERIAL**

S1: SUMMARY OF COGNITIVE DEBRIEFING FINDINGS

S2: CONFIRMATORY FACTOR ANALYSIS MODEL PLOTS

S3: SELF-CARE IN CHRONIC OBSTRUCTIVE PULMONARY DISEASE INVENTORY (PORTUGUESE VERSION)

S4: SELF-CARE IN CHRONIC OBSTRUCTIVE PULMONARY DISEASE INVENTORY (ORIGINAL INSTRUMENT) (Matarese et al., 2020) available at:

<https://self-care-measures.com/project/patient-version-sccopd-english-2/>

**S1: SUMMARY OF COGNITIVE DEBRIEFING FINDINGS**

As part of the cognitive debriefing phase, the translated instrument was administered to 20 native Portuguese-speaking participants aged between 55 and 86 years. This group was intentionally selected to reflect a range of socioeconomic backgrounds, educational levels, and health statuses, including both individuals with and without chronic or respiratory conditions. The objective of this phase was to assess the clarity, cultural appropriateness, and overall comprehension of the translated items.

In general, participants were able to understand the purpose of the instrument and articulate the meaning of most items without difficulty. However, a few terms and phrasings prompted questions or suggestions for improvement, which are summarized below:

**1. Terminology: “Exacerbação” (Exacerbation)**

A significant number of participants (12 out of 20) expressed confusion or uncertainty about the term *exacerbação*, commonly used in clinical settings to describe a worsening of symptoms. When probed further, many reported that they were either unfamiliar with the term or did not associate it with respiratory conditions. In contrast, the word *crise* (crisis) was immediately recognized and understood by all participants as referring to an episode of symptom worsening, particularly in the context of chronic illness.

**Suggested revision:** Replace *exacerbação* with *crise/exacerbação* to improve clarity and ensure broader understanding across populations with varying health literacy levels.

**2. Inclusion of “Enfermeiro” (Nurse) alongside “Médico” (Doctor)**

When responding to items referring to communication with healthcare professionals, 9 out of 20 participants noted that, in their experience, they often discuss symptoms or health concerns with a nurse, particularly in cases where they receive ongoing care for chronic conditions such as diabetes or hypertension. In Portugal, it is common for patients to be followed by specialized nurses who play an active role in disease management and patient education. For this reason, participants suggested that referencing only a *médico* (doctor) did not fully reflect their healthcare experience.

**Suggested revision:** Modify the relevant item(s) to read *enfermeiro ou médico* (nurse or doctor) to acknowledge the broader spectrum of professional care providers involved in chronic disease management.

These suggestions were submitted to the original author of the instrument for review and were taken into consideration during the finalization phase of the translation process.

**S2: CONFIRMATORY FACTOR ANALYSIS MODEL PLOTS**

**SELF-CARE MAINTENANCE**


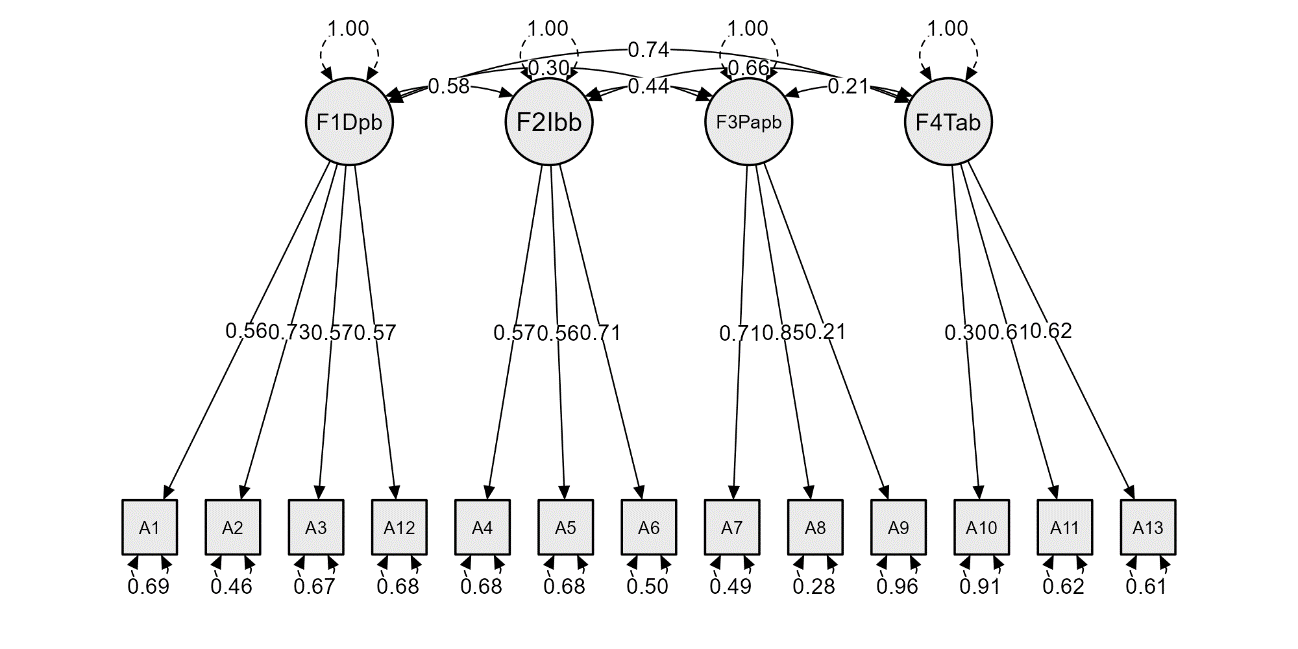


**SELF-CARE MONITORING**


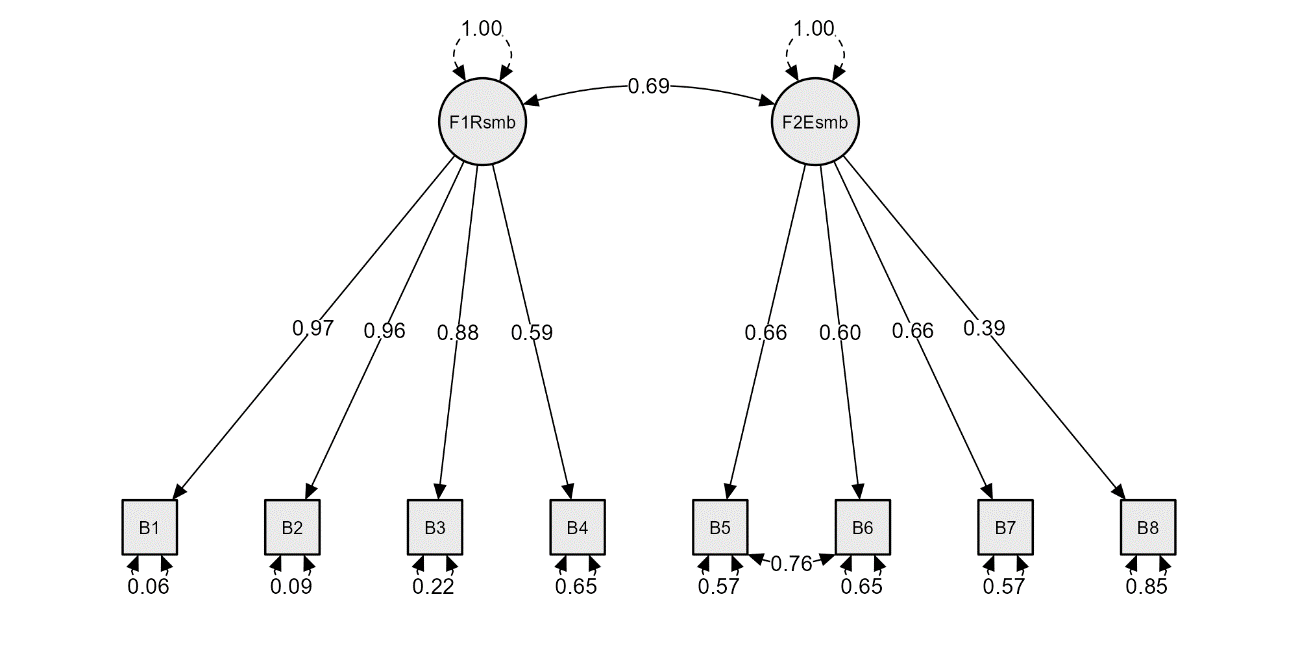


**SELF-CARE MANAGEMENT**


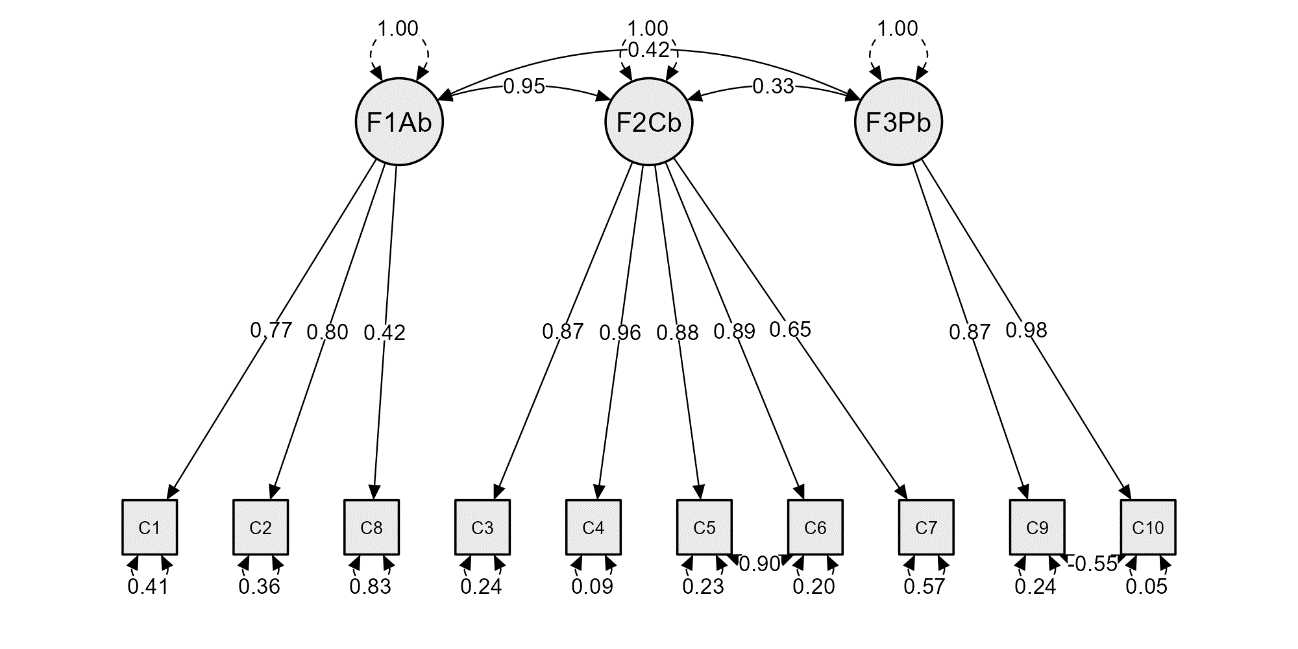


**SELF-CARE SELF-EFFICACY**


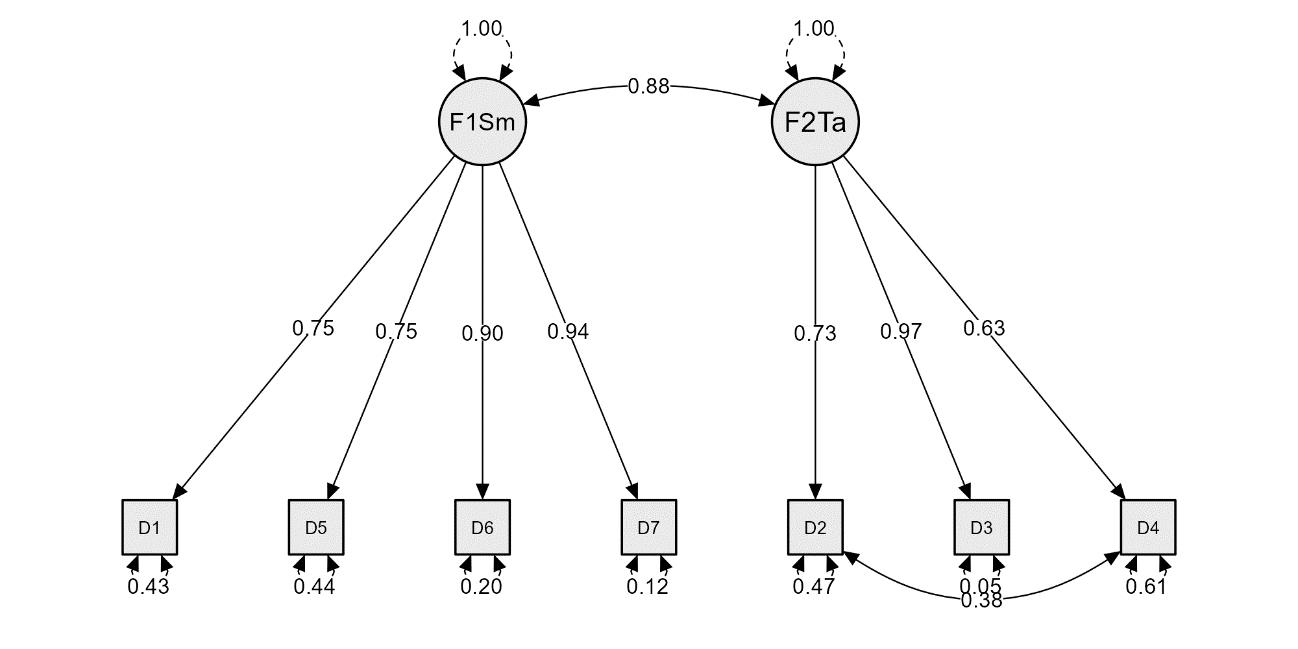


**S3: SELF-CARE IN CHRONIC OBSTRUCTIVE PULMONARY DISEASE INVENTORY (PORTUGUESE VERSION)**

**INVENTÁRIO DO AUTOCUIDADO NA PESSOA COM DOENÇA PULMONAR OBSTRUTIVA CRÓNICA (IA-DPOC)**

**SECÇÃO A**

Abaixo estão identificados comportamentos comuns que as pessoas com doenças pulmonares crónicas apresentam para preservar a sua saúde e bem-estar. Por favor indique com que frequência é que adota os seguintes comportamentos:

|  | | | NUNCA | RARAMENTE | ÀS VEZES | FREQUENTEMENTE | SEMPRE |
| --- | --- | --- | --- | --- | --- | --- | --- |
| 1 | Evito pessoas com constipações ou gripes | | 1 | 2 | 3 | 4 | 5 |
| 2 | Afasto-me da sala/local onde alguém está a fumar | | 1 | 2 | 3 | 4 | 5 |
| 3 | Evito o contacto com sprays, tintas, solventes e pó | | 1 | 2 | 3 | 4 | 5 |
| 4 | Mantenho os meus pulmões limpos tossindo ou com respirações profundas se necessário | | 1 | 2 | 3 | 4 | 5 |
| 5 | Faço pausas durante as minhas atividades diárias para descansar | | 1 | 2 | 3 | 4 | 5 |
| 6 | Uso a respiração abdominal ou com lábios semicerrados para controlar a minha respiração | | 1 | 2 | 3 | 4 | 5 |
| 7 | Faço regularmente algum tipo de exercício (caminhada, ciclismo, natação, etc.) | | 1 | 2 | 3 | 4 | 5 |
| 8 | Exercito os meus braços pelo menos 3 vezes por semana | | 1 | 2 | 3 | 4 | 5 |
| 9 | Participo em atividades sociais com outras pessoas pelo menos uma vez por semana | | 1 | 2 | 3 | 4 | 5 |
| 10 | Tomo a vacina da gripe todos os anos | | 1 | 2 | 3 | 4 | 5 |
| 11 | Tomo a medicação tal como prescrita pelo meu médico | NÃO TENHO PRESCRIÇÕES DE MEDICAÇÃO | 1 | 2 | 3 | 4 | 5 |
| 12 | Protejo a boca/nariz quando estou fora de casa e o ar está frio | | 1 | 2 | 3 | 4 | 5 |
| 13 | Faço consultas regulares com o meu médico para realizar exames relacionados com a doença pulmonar crónica | | 1 | 2 | 3 | 4 | 5 |

**SECÇÃO B**

Abaixo estão identificados comportamentos comuns que as pessoas com doenças pulmonares crónicas adotam para avaliar a sua doença. Indique com que frequência adota os seguintes comportamentos:

NA=NÃO SE APLICA A MIM/ NÃO APLICÁVEL

|  | | EU NÃO TENHO ESTE PROBLEMA | NUNCA | RARAMENTE | ÀS VEZES | FREQUENTEMENTE | SEMPRE |
| --- | --- | --- | --- | --- | --- | --- | --- |
| 1 | Verifico se existe um aumento na quantidade de expetoração | NA | 1 | 2 | 3 | 4 | 5 |
| 2 | Verifico se existe uma alteração na cor da expetoração | NA | 1 | 2 | 3 | 4 | 5 |
| 3 | Verifico se existe um aumento na tosse | NA | 1 | 2 | 3 | 4 | 5 |
| 4 | Verifico se existe um aumento na falta de ar ou pieira ("chiadeira") | NA | 1 | 2 | 3 | 4 | 5 |
| 5 | Verifico se acordo durante a noite com dificuldade em respirar | NA | 1 | 2 | 3 | 4 | 5 |
| 6 | Verifico se tenho dificuldade em adormecer devido à dificuldade em respirar | NA | 1 | 2 | 3 | 4 | 5 |
| 7 | Verifico se me canso mais do que o habitual quando faço alguma coisa | NA | 1 | 2 | 3 | 4 | 5 |
| 8 | Verifico se tenho palpitações, tremores, insónia, boca seca e dificuldade em urinar após fazer os inaladores | EU NÃO FAÇO INALADORES | 1 | 2 | 3 | 4 | 5 |

9. Pessoas com doenças pulmonares crónicas podem ter sintomas devido à sua doença ou ao tratamento que estão a receber para a doença. Da **última vez** que teve sintomas, com que rapidez o reconheceu como sintoma da sua doença?

| EU NÃO TIVE SINTOMAS | EU NÃO RECONHECI O SINTOMA | NÃO IMEDIATAMENTE |  | QUASE IMEDIATAMENTE |  | IMEDIATAMENTE |
| --- | --- | --- | --- | --- | --- | --- |
| NA | 0 | 1 | 2 | 3 | 4 | 5 |

**SECÇÃO C**

Abaixo estão identificados comportamentos comuns que as pessoas com doença pulmonar crónica adotam para gerir os seus sintomas. Indique a probabilidade de adotar um dos seguintes comportamentos se tiver sintomas.

NA= Não se aplica a mim

|  | | | IMPROVÁVEL |  | PROVAVELMENTE |  | MUITO PROVAVELMENTE |
| --- | --- | --- | --- | --- | --- | --- | --- |
| 1 | Falo com o meu médico ou enfermeiro se tiver problemas com a medicação para a doença pulmonar crónica | EU NÃO TOMO MEDICAÇÃO | 1 | 2 | 3 | 4 | 5 |
| 2 | Recorro ao meu médico ou enfermeiro se apresentar algum problema de saúde que dure mais do que alguns dias | | 1 | 2 | 3 | 4 | 5 |
| 3 | Falo com o meu médico ou enfermeiro se sentir que a falta de ar aumentou | | 1 | 2 | 3 | 4 | 5 |
| 4 | Falo com o meu médico ou enfermeiro se sentir que a tosse aumentou | NA | 1 | 2 | 3 | 4 | 5 |
| 5 | Falo com o meu médico ou enfermeiro se a expetoração mudar de cor | NA | 1 | 2 | 3 | 4 | 5 |
| 6 | Falo com o meu médico ou enfermeiro se a quantidade de expetoração aumentar | NA | 1 | 2 | 3 | 4 | 5 |
| 7 | Falo com o meu médico ou enfermeiro se tiver efeitos secundários dos inaladores (por exemplo, tremores, insónia, boca seca, dificuldade em urinar) | EU NÃO FAÇO INALADORES | 1 | 2 | 3 | 4 | 5 |
| 8 | Quando os sintomas da minha doença pioram, eu altero a medicação prescrita de acordo com o que o meu médico me indicou (por exemplo, tomar cortisona e/ou um antibiótico) | EU NÃO TOMO MEDICAÇÃO | 1 | 2 | 3 | 4 | 5 |
| 9 | Sento-me enquanto realizo tarefas domésticas quando sinto falta de ar | | 1 | 2 | 3 | 4 | 5 |
| 10 | Quando tomo banho e sinto falta de ar, sento-me numa cadeira ou outro suporte. | | 1 | 2 | 3 | 4 | 5 |

**AUTOEFICÁCIA NO AUTOCUIDADO**

| Indique o grau de confiança que sente na sua capacidade de realizar as  atividades identificadas abaixo. | | NÃO TENHO CONFIANÇA |  | TENHO CONFIANÇA |  | TENHO MUITA CONFIANÇA |
| --- | --- | --- | --- | --- | --- | --- |
| 1 | Prevenir o aparecimento de sintomas da minha doença pulmonar crónica | 1 | 2 | 3 | 4 | 5 |
| 2 | Seguir o conselho terapêutico que me deram, mesmo quando é complicado | 1 | 2 | 3 | 4 | 5 |
| 3 | Continuar a verificar os meus sintomas, mesmo que seja complicado | 1 | 2 | 3 | 4 | 5 |
| 4 | Tomar a medicação corretamente, seguindo as instruções dadas, mesmo que seja complicado | 1 | 2 | 3 | 4 | 5 |
| 5 | Reconhecer os sintomas de uma crise/exacerbação da doença pulmonar crónica quando eles aparecem | 1 | 2 | 3 | 4 | 5 |
| 6 | Fazer alguma coisa para aliviar os sintomas, mesmo que seja difícil | 1 | 2 | 3 | 4 | 5 |
| 7 | Avaliar se os comportamentos adotados para aliviar os sintomas foram eficazes | 1 | 2 | 3 | 4 | 5 |

**S4: SELF-CARE IN CHRONIC OBSTRUCTIVE PULMONARY DISEASE INVENTORY (ORIGINAL INSTRUMENT)**

**SELF-CARE IN CHRONIC OBSTRUCTIVE PULMONARY DISEASE INVENTORY (SC-COPDI)**

**SECTION A**

Listed below are common behaviors that people suffering from chronic lung diseases do to maintain their health and well-being. Please indicate how often you perform the following behaviors:

|  | | | NEVER | RARELY | SOMETIMES | OFTEN | ALWAYS |
| --- | --- | --- | --- | --- | --- | --- | --- |
| 1 | Avoid people with colds or flu | | 1 | 2 | 3 | 4 | 5 |
| 2 | Move away from the room / place where someone is smoking | | 1 | 2 | 3 | 4 | 5 |
| 3 | Avoid contact with sprays, paints, solvents and dust | | 1 | 2 | 3 | 4 | 5 |
| 4 | Keep my lungs free by coughing or with deep breathing if needed | | 1 | 2 | 3 | 4 | 5 |
| 5 | Pause during my daily activities to rest | | 1 | 2 | 3 | 4 | 5 |
| 6 | Use abdominal breathing or pursed lips breathing to regulate my breath | | 1 | 2 | 3 | 4 | 5 |
| 7 | Regularly do some form of exercise (walking, cycling, swimming, etc.) | | 1 | 2 | 3 | 4 | 5 |
| 8 | Exercise with my arms at least 3 times a week | | 1 | 2 | 3 | 4 | 5 |
| 9 | Engage in social activities with other people at least once a week | | 1 | 2 | 3 | 4 | 5 |
| 10 | Get a flu vaccination every year | | 1 | 2 | 3 | 4 | 5 |
| 11 | Take the medicines as prescribed by my healthcare provider | I DO NOT HAVE  MEDICINE PRESCRIPTION | 1 | 2 | 3 | 4 | 5 |
| 12 | Protect my mouth/nose when I walk outdoors and the air is cold | | 1 | 2 | 3 | 4 | 5 |
| 13 | Make regular visits to my healthcare provider for checks-ups of my chronic lung disease | | 1 | 2 | 3 | 4 | 5 |

**SECTION B**

Listed below are common behaviors that people with chronic lung diseases can perform to monitor their disease. Indicate how often you perform the following behaviors:

|  | | I DO NOT HAVE SUCH TROUBLE | NEVER | RARELY | SOMETIMES | OFTEN | ALWAYS |
| --- | --- | --- | --- | --- | --- | --- | --- |
| 1 | Monitor for an increase in sputum quantity | NA | 1 | 2 | 3 | 4 | 5 |
| 2 | Monitor for a change in sputum color | NA | 1 | 2 | 3 | 4 | 5 |
| 3 | Monitor for an increase of coughing | NA | 1 | 2 | 3 | 4 | 5 |
| 4 | Monitor for an increase in breathlessness or whistles | NA | 1 | 2 | 3 | 4 | 5 |
| 5 | Monitor whether I wake up during the night with trouble breathing | NA | 1 | 2 | 3 | 4 | 5 |
| 6 | Check whether I struggle to fall asleep due to trouble breathing | NA | 1 | 2 | 3 | 4 | 5 |
| 7 | Monitor whether I get tired more than usual when I do something | NA | 1 | 2 | 3 | 4 | 5 |
| 8 | Check for palpitations, tremor, insomnia, dry mouth and difficulty at urinating after taking inhaled medications | I DO NOT TAKE INHALATORS | 1 | 2 | 3 | 4 | 5 |

NA=IT DOES NOT APPLY TO ME

9. People with chronic lung diseases can have symptoms due to their illness or to the treatment they are receiving for their illness. The **last time** you had symptoms, how quickly did you recognize it as a symptom of your illness?

| I HAVE NOT HAD SYMPTOMS | I DID NOT RECOGNIZE THE SYMPTOM | NOT QUICKLY |  | SOMEWHAT QUICKLY |  | VERY QUICKLY |
| --- | --- | --- | --- | --- | --- | --- |
| NA* | 0 | 1 | 2 | 3 | 4 | 5 |

* Do not fill in section C in case you have never had symptoms.

**SECTION C**

Listed below are common behaviors that people with chronic lung disease perform to manage their symptoms. Indicate how likely you are to perform one of following behaviors when you have symptoms.

|  | | | NOT LIKELY |  | SOMEWHAT LIKELY |  | VERY LIKELY |
| --- | --- | --- | --- | --- | --- | --- | --- |
| 1 | Talk to my healthcare provider if I have problems with prescriptions for my chronic lung disease | I DO NOT TAKE  MEDICINES | 1 | 2 | 3 | 4 | 5 |
| 2 | Go to my healthcare provider if I have any health problem that lasts for more than a few days | | 1 | 2 | 3 | 4 | 5 |
| 3 | Speak to my healthcare provider if I feel that the breathlessness has increased | | 1 | 2 | 3 | 4 | 5 |
| 4 | Speak to my healthcare provider if I feel that the cough has increased | NA | 1 | 2 | 3 | 4 | 5 |
| 5 | Speak to my healthcare provider if the sputum changes color | NA | 1 | 2 | 3 | 4 | 5 |
| 6 | Speak to my healthcare provider if the amount of sputum increases | NA | 1 | 2 | 3 | 4 | 5 |
| 7 | Speak to my healthcare provider if I get side effects from my inhaled medicines (e.g., tremor, insomnia, dry mouth, difficulty urinating) | I DO NOT TAKE INHALATORS | 1 | 2 | 3 | 4 | 5 |
| 8 | When the symptoms of my illness worsen, I modify prescribed therapy as my healthcare provider told me to do (for example, take cortisone and/or an antibiotic) | I DO NOT TAKE  MEDICINES | 1 | 2 | 3 | 4 | 5 |
| 9 | Sit doing housework when I have breathlessness | | 1 | 2 | 3 | 4 | 5 |
| 10 | When I have breathlessness, sit on a chair or on another support when I shower or use the bathtub | | 1 | 2 | 3 | 4 | 5 |

NA=IT DOES NOT APPLY TO ME

**SELF-CARE-SELF-EFFICACY**

Indicate how much confidence you feel in your ability to carry out the activities listed below.

|  | | NOT CONFIDENT |  | SOMEWHAT CONFIDENT |  | EXTREMELY CONFIDENT |
| --- | --- | --- | --- | --- | --- | --- |
| 1 | Prevent the onset of symptoms of my chronic lung disease | 1 | 2 | 3 | 4 | 5 |
| 2 | Follow the therapeutic advice they gave me, even if it's difficult | 1 | 2 | 3 | 4 | 5 |
| 3 | Continue to check my symptoms even if it's not always easy | 1 | 2 | 3 | 4 | 5 |
| 4 | Take medicines properly, following the instructions given even if it difficult | 1 | 2 | 3 | 4 | 5 |
| 5 | Recognize the symptoms of an exacerbation of chronic lung disease when they appear | 1 | 2 | 3 | 4 | 5 |
| 6 | Do something to relieve symptoms, even if it is difficult | 1 | 2 | 3 | 4 | 5 |
| 7 | Assess whether the behaviors performed to relieve the symptoms have been effective | 1 | 2 | 3 | 4 | 5 |
